# Letermovir prophylaxis for cytomegalovirus in lung-transplant recipients: a comprehensive study with literature review of off-label use and real-world experiences

**DOI:** 10.1007/s10238-024-01330-2

**Published:** 2024-04-05

**Authors:** Takashi Hirama, Yuki Shundo, Toshikazu Watanabe, Akihiro Ohsumi, Tatsuaki Watanabe, Yoshinori Okada

**Affiliations:** 1 Department of Thoracic Surgery, Institute of Development, Aging and Cancer, Tohoku University, Sendai, Miyagi Japan; 2 Division of Organ Transplantation, Tohoku University Hospital, Sendai, Miyagi Japan; 3 Department of Respiratory Medicine, Faculty of Medicine, Fukuoka University, Fukuoka, Fukuoka Japan; 4 Department of Thoracic Surgery, Kyoto University Hospital, Kyoto, Kyoto Japan

**Keywords:** Lung transplant, Letermovir, Cytomegalovirus, Prophylaxis, Off-label use

## Abstract

Letermovir, initially approved for cytomegalovirus (CMV) prophylaxis in hematopoietic stem-cell transplantation, has gained attention for off-label use in lung-transplant (LTx) recipients. Given the high susceptibility of LTx recipients to CMV infection, this study explores the effectiveness and safety of letermovir prophylaxis. A retrospective analysis of using letermovir for LTx recipients at Tohoku University Hospital (January 2000 to November 2023) was conducted. Case summaries from other Japanese transplant centers and a literature review were included. Six cases at Tohoku University Hospital and one at Kyoto University Hospital were identified. Prophylactic letermovir use showed positive outcomes in managing myelosuppression and preventing CMV replication. The literature review supported the safety of letermovir in high-risk LTx recipients. Despite limited reports, our findings suggest letermovir’s potential as prophylaxis for LTx recipients intolerant to valganciclovir. Safety, especially in managing myelosuppression, positions letermovir as a promising option. However, careful consideration is important in judiciously integrating letermovir into the treatment protocol.

## Introduction

Letermovir, approved by the U.S. Food and Drug Administration (FDA) in November 2017 for cytomegalovirus (CMV) prophylaxis post-allogeneic hematopoietic stem-cell transplantation (HSCT), has gained global recognition since its subsequent approvals in Europe in January 2018 and Japan in March 2018. Following the outcomes of a Phase III study involving kidney transplant (KTx) recipients [1], the FDA expanded letermovir’s indications for cytomegalovirus (CMV) prophylaxis to encompass adult KTx recipients at high risk, especially with respect to CMV IgG-positive donors/CMV IgG-negative recipients (D + /R −). The study underscores the importance of extending letermovir’s approval to lung-transplant (LTx) recipients, a group facing a substantial risk of CMV infection, often complicated by ganciclovir-resistant CMV [2, 3]. This research scrutinizes the off-label use of letermovir for CMV prophylaxis in LTx recipients at our transplant center, Tohoku University Hospital (TUH), particularly those exhibiting valganciclovir intolerance. Additionally, we present a compilation of literature-based reports from various other transplant centers on the use of letermovir for LTx recipients.

## Methods

### Off-label use of letermovir

Patients who underwent LTx at TUH from January 2000 to November 2023 were retrospectively analyzed to determine the utilization of letermovir between 2019 and 2023. Letermovir, available for prescription at TUH for HSCT recipients since January 2019, underwent rigorous evaluation by the Pharmaceutical Safety Management Office at TUH and gained approval for off-label use for LTx recipients in July 2019 (19–010).

The prescription of letermovir necessitates obtaining additional approvals, which includes the submission of patient-specific drug use applications to both the Pharmaceutical Department at TUH and the Health Insurance Claims Review and Reimbursement Service of the local government. This rigorous process ensures a meticulous focus on safety and addresses the individual needs of each patient.

As of the end of November 2023, insights were sought from practitioners at all of Japan’s 11 lung-transplant centers to gather information on their experiences in the use of letermovir in LTx recipients. These are detailed in the acknowledgments.

### Management of CMV and immunosuppression

Post-LTx CMV management are implemented based on CMV serology at TUH. For the seronegative donor (D −) and the seronegative recipient (R −), no prophylaxis against CMV is administered, and valaciclovir 500 mg is administered to address herpes simplex virus and varicella-zoster virus infections for three months post-LTx. For recipients who are seropositive (D +) regardless of the donor status, valganciclovir 900 mg is administered for the first three months post-LTx, followed by administration three times weekly up to 12 months post-LTx. In cases of CMV serology mismatch (D + /R −), administration of valganciclovir 900 mg is continued for 12 months post-LTx.

To manage myelosuppression, reduction of valganciclovir is considered for CMV D + /R + or D − /R + , while a reduction in mycophenolic mofetil is considered for CMV mismatch cases. If myelosuppression persists despite dose reduction or discontinuation of both drugs, letermovir 480 mg is considered as an alternative to valganciclovir, and a low dosage of mycophenolic mofetil is continued. For CMV detection post-LTx, a pp65 antigenemia assay was used until March 2023, followed by the adoption of nucleic acid amplification tests from April 2023 onwards. CMV screening was performed at different intervals: twice weekly during the initial postoperative phase (ICU stay), weekly from ICU discharge until hospital release, and monthly throughout the first year after transplantation. The definitions of CMV infection and CMV disease adhere to guidelines [2].

All LTx recipients are required to adhere to a lifelong maintenance regimen comprising tacrolimus, mycophenolic mofetil, and prednisolone. The adjustment of dosages has been documented in prior reports [4]. In situations where tacrolimus or mycophenolic mofetil cannot be tolerated, cyclosporine or azathioprine, respectively, is substituted [5]. Prophylaxis involving antimicrobials not confined to CMV [6–8], histocompatibility testing [5], and the comprehensive management of LTx recipients [9, 10] have been previously reported. The overall post-transplantation management is comparable between TUH and Kyoto University Hospital (KUH). However, TUH utilizes voriconazole for universal prophylaxis, whereas KUH employs itraconazole [11].

### Literature review

A systematic search of the PubMed database and Google Scholar using the terms “letermovir” and “lung transplant” was conducted for the period between January 2016 and November 2023. Our focus was on citing case series involving the use of letermovir in LTx recipients, which comprise compilations in two or more individual cases. To avoid duplication, we prioritized citing the latest report from any transplant center that published multiple reports on letermovir use in LTx recipients.

### Results

Between January 2000 and November 2023, our retrospective analysis at TUH identified six LTx recipients who had been administered letermovir. Collaboration with other Japanese LTx centers revealed an additional case at KUH. The characteristics of the seven recipients are summarized in Table 1.

**Table 1:** Characteristics of lung-transplant recipients taking letermovir—institutional cases

	Case 1	Case 2	Case 3	Case 4	Case 5	Case 6	Case 7
Conditions BEFORE letermovir use							
Age at LTx	57	49	59	42	31	39	29
Sex, female	X		X				
*LTx procedure*							
Single LTx	X	X	X		X		
Bilateral LTx				X		X	X
Living-donor LTx							
Multi-organ transplant							
*LTx indication*							
Obstructive							X
Vascular				X			
Suppurative							
Fibrosis	X	X	X				
Allogeneic					X	X	
History of any transplantation prior to LTx					X	X	
*CMV serology*							
D − /R −							
D + /R −				X		X	X
D − /R +							
D + /R +	X	X	X		X		
History of acute cellular/humoral rejection			X				
*Maintenance immunosuppression*							
FK-506 + MMF + prednisone			X			X	X
FK-506 + low MMF + prednisone	X						
FK-506 + prednisone		X					
FK-506 + AZA + prednisone				X	X		
History of CMV infection	X	X	X				X
History of any CMV disease			X				X
WBC/neutrophil nadir (cell/uL) prior to letermovir use	2700/1680	200/10	1300/1120	1500/1170	600/220	1500/890	4250/2140
Conditions AFTER letermovir use							
*Indication for letermovir*							
Primary prophylaxis				X	X	X	
Secondary prophylaxis	X	X	X				X
Treatment for CMV infection/disease							
Time from LTx to letermovir use (months)	2	4	34	1	1	1	4
*Rationale for letermovir use*							
Myelosuppression	X	X	X	X	X	X	
Ganciclovir-resistant CMV/UL97							X
Refractory CMV disease							
Duration of letermovir use (months)	26	32	5	12	1	2	4
Adverse event due to letermovir							
Disseminated VZV/HSV infection		X					
CMV infection or disease while receiving letermovir		X					
Letermovir-resistant CMV/UL56							
Continued letermovir use at time of data analysis (Nov 2023)					X	X	X

LTx, lung transplant; D, donor; R, recipient; + seropositive; − seronegative; FK-506, tacrolimus; MMF, mycophenolic mofetil; CMV, cytomegalovirus; VZV, varicella-zoster virus; HSV, herpes simplex virus; WBC, white blood cell

Case 1: A 57-year-old male underwent right single LTx for progressive pleuroparenchymal fibroelastosis (PPFE). The CMV serology indicated D + /R + . The perioperative period was intricate, marked by recurrent pneumothorax in the native lung on the left, a right femoral neck fracture subsequent to a fall, painful peripheral neuropathy attributed to voriconazole, acute kidney injury, and myelosuppression resulting from valganciclovir. Because of a neutrophil count dropping to 1680, mycophenolate mofetil was reduced, and the use of valganciclovir was discontinued two months post-LTx. CMV replication was confirmed once by CMV antigenemia, which promptly resolved. Following the approval of a patient-specific drug use application, secondary prophylaxis with letermovir was initiated. The patient has not experienced acute rejection post-LTx, maintaining stable graft function up to the current two-year mark. Various complications other than graft have also subsided, and letermovir was discontinued at 26 months post-transplant. Subsequent monitoring has not revealed CMV replication.

Case 2: A 49-year-old female underwent right single LTx for fibrotic lung disease following severe community-acquired pneumonia. The CMV serology indicated D + /R + . She suffered from right phrenic nerve paralysis during the perioperative period, necessitating 29 days of mechanical ventilation, followed by non-invasive positive pressure ventilation (NPPV) for one-year post-LTx. Severe neutropenia induced by either mycophenolate mofetil or valganciclovir led to the discontinuation of both medications, requiring lenograstim administration. Minimal CMV replication was detected a few times through CMV antigenemia testing while in the intensive care unit (ICU). The introduction of letermovir prompted a modification in the immunosuppressive regimen, maintaining a two-drug combination of calcineurin inhibitors and corticosteroids without metabolic antagonists. Three months after letermovir was initiated, she developed disseminated varicella and was treated with acyclovir. Despite various complications and CMV detection even after letermovir initiation, letermovir was ultimately discontinued three years post-LTx, and subsequent follow-up revealed no further CMV replication. Graft function remains stable, and the patient sustains a high quality of life without the need for NPPV.

Case 3: A 59-year-old male underwent a left single LTx for idiopathic pulmonary fibrosis, with a medical history of ulcerative colitis. The CMV serology revealed D + /R + . Despite recurrent minimal CMV replication detected through CMV antigenemia testing, the patient had been on long-term prophylactic treatment with valganciclovir and no history of any symptoms of CMV. Acute rejection occurred two years post-LTx, necessitating steroid pulse therapy. Subsequently, high-dose mycophenolate mofetil was administered, leading to myelosuppression. Valganciclovir was discontinued, and letermovir was substituted, resulting in subsequent neutrophil count stabilization (> 1500) and no further detection of CMV. The patient later succumbed to colonic perforation, but histopathological examination revealed no evidence of CMV disease.

Case 4: A 42-year-old female underwent bilateral LTx for idiopathic pulmonary arterial hypertension. Because of her medical history of Graves’ disease and paroxysmal atrial fibrillation, left atrial appendage excision was performed during the LTx surgery for thromboprophylaxis. Postoperatively, she experienced a severe esophageal injury, resulting in an extended 61-day stay in the ICU. Despite CMV mismatch (D + /R −), no evidence of CMV replication was detected by PCR. Thirty days after LTx, she developed myelosuppression. Given the CMV mismatch, valganciclovir was continued, and mycophenolate mofetil was gradually reduced. However, with a persistent decline in blood cell counts, a final adjustment was made to a low dosage of azathioprine. Despite this, hematopoietic recovery was not observed, prompting the initiation of letermovir. Subsequent monitoring revealed no CMV replication. Graft function remains stable, and the patient maintains a high quality of life. As per the protocol, CMV prophylaxis was discontinued 12 months after transplantation. At 6 months after the cessation of letermovir, CMV replication has not been observed.

Case 5: A 31-year-old female, previously diagnosed with Hodgkin lymphoma, underwent HSCT ten years ago. Recently, she underwent a right single LTx because of chronic graft-versus-host disease (GVHD). The CMV serology indicated D + /R + , and there was no previous history of CMV infection or disease during the allogeneic HSCT. The LTx procedure produced no complications, and standard immunosuppressive therapy was begun. However, one month post-LTx, she exhibited a gradual onset of myelosuppression. Attempts to reduce mycophenolate mofetil and valganciclovir resulted in a decline in the neutrophil count to 220, leading to the discontinuation of both medications and the administration of lenograstim for three days. Following confirmation of the absence of hematological toxicity with azathioprine, letermovir was initiated. Letermovir is currently being administered and is planned to be continued for up to one year post-LTx according to the protocol at TUH.

Case 6: A 38-year-old female underwent living-donor LTx for systemic sclerosis-associated interstitial lung disease. However, because of stenosis at the pulmonary vein anastomosis, she underwent deceased-donor LTx one year later. In the initial LTx, the CMV serology was D − /R − , and in accordance with our protocol, no prophylaxis for CMV was administered. In the second LTx, a CMV serology mismatch (D + /R −) prompted the initiation of valganciclovir. The immunosuppressive protocol remained consistent between the first and second LTx, with no changes in medications or dosages. One month after LTx, neutropenia was observed, leading to the discontinuation of valganciclovir and the initiation of letermovir, resulting in subsequent improvement in the neutrophil count. Since the first LTx, no evidence of CMV replication has been detected. Lung function remains stable, and it is planned to continue administering letermovir for one year post-LTx.

Case 7: This case was handled by Kyoto University. A 29-year-old female underwent bilateral LTx for obstructive lung disease associated with cutis laxa. Her CMV serology demonstrated D + /R − mismatch. Three months post-LTx, she developed CMV disease, which was refractory to ganciclovir and thus treated with foscarnet and reduction in immunosuppressive medications, leading to undetectable CMV replication. Subsequently, the CMV profile exhibited resistance to ganciclovir. Consequently, letermovir was initiated four months post-LTx as secondary prophylaxis. She has since been able to continue receiving letermovir without any adverse events.

## Literature review

As of November 2023, four articles have documented the use of letermovir for LTx recipients, all in the form of a case series. Combining experiences from Japan, a comprehensive summary of five reports is presented in Table 2.

**Table 2:** Characteristics of lung-transplant recipients taking letermovir—literature review

Years of letermovir use	2018–2019	2018–2020	2018	2022–2023	2018–2020
Study region, Country	North Carolina, USA	Ohio, USA	Virginia, USA	Sendai, Japan	Munich, Germany
Number of recipients	*n* = 41	*n* = 17	*n* = 8	*n* = 7	*n* = 28
Conditions BEFORE letermovir use					
Median age (IQR) or mean age ± SD	53 (range 23–76)	62 (IQR 54–64)	63 (IQR 59–67)	42 (IQR 35–53)	53.7 ± 8.5 SD
Sex, female, *n* (%)	16 (43.2%)	7 (41.2%)	6 (75%)	2 (28.6%)	15 (53.6%)
*LTx procedure, n (%)*					
Single	5 (13.5%)	2 (11.8%)	3 (37.5%)	4 (57.1%)	
Bilateral	32 (86.5%)	15 (88.2%)	5 (62.5%)	3 (42.9%)	
Living-donor	0 (0.0%)	0 (0.0%)	0 (0.0%)	0 (0.0%)	
Multi-organ	0 (0.0%)	0 (0.0%)	0 (0.0%)	0 (0.0%)	
Heart	4 (10.8%)				
*LTx indication, n (%)*					
Obstructive	7 (18.9%)	4 (23.5%)	2 (25%)	1 (14.3%)	13 (46.4%)
Vascular	0 (0.0%)	0 (0.0%)	1 (12.5%)	1 (14.3%)	0 (0.0%)
Suppurative	12 (32.4%)	4 (23.5%)	1 (12.5%)	0 (0.0%)	4 (14.3%)
Fibrosis	14 (37.8%)	7 (41.2%)	4	3 (42.9%)	10 (35.7%)
Allogeneic	0 (0.0%)	1 (5.9%)	0 (0.0%)	2 (28.6%)	0 (0.0%)
Others	8 (21.6%)	1 (5.9%)	0 (0.0%)		1 (3.6%)
History of any transplantation prior to LTx	6 (16.2%)			2 (28.6%)	
*CMV serology*					
D − /R −	1 (2.7%)	0 (0.0%)	0 (0.0%)	0 (0.0%)	0 (0.0%)
D + /R −	21 (56.8%)	11 (64.7%)	6 (75%)	3 (42.9%)	15 (53.6%)
D − /R +	8 (21.6%)	4 (23.5%)	1 (12.5%)	0 (0.0%)	0 (0.0%)
D + /R +	10 (27.0%)	2 (11.8%)	1 (12.5%)	4 (57.1%)	3 (10.7%)
History of acute cellular/humoral rejection				1 (14.3%)	3 (10.7%)
*Maintenance immunosuppression*					
FK-506 + MMF + prednisone			2 (25%)	3 (42.9%)	14 (50.0%)
FK-506 + low MMF + prednisone			1 (12.5%)	1 (14.3%)	5 (17.9%)
FK-506 + prednisone			4 (50%)	1 (14.3%)	0 (0.0%)
FK-506 + AZA + prednisone			0 (0.0%)	2 (28.6%)	0 (0.0%)
Cyclosporine + MMF + prednisone			0 (0.0%)		4 (14.3%)
Cyclosporine + prednisone			1 (12.5%)		0 (0.0%)
FK-506 + low MMF + prednisone + mTOR			0 (0.0%)		5 (17.9%)
History of CMV infection		8 (47.1%)	6 (75%)	4 (57.1%)	28 (100%)
History of any CMV disease			1 (12.5%)	2 (28.6%)	15 (53.6%)
WBC nadir (cell/uL) prior to letermovir use		1100 (range 900–1300)		1500 (IQR 950–2100)	
Conditions AFTER letermovir use					
*Indication for CMV prophylaxis with letermovir*					
Primary prophylaxis	26 (70.3%)	9 (52.9%)	7 (87.5%)	5 (71.4%)	0 (0.0%)
Secondary prophylaxis	16 (43.2%)*2	8 (47.1%)	1 (12.5%)	2 (28.6%)	0 (0.0%)
Treatment for CMV infection/disease	0 (0.0%)	0 (0.0%)	1 (12.5%)*1	0 (0.0%)	28 (100%)
Time from LTx to letermovir use (months)	PP 10.4 (IQR 4.1–37.4) SP 22.8 (IQR 17.6–38.0)	6.1 (range 2.5–25.2)	10 (IQR 7–15)	2 (IQR 1–4)	20.7 ± 26.2
*Rationale for letermovir use*					
Myelosuppression	PP 25 (96.2%) SP 10 (62.5%)	17 (100%)	7 (87.5%)	6 (86.7%)	
Ganciclovir-resistant CMV/UL97	PP 0 (0.0%) SP 6 (37.5%)		1 (12.5%)	1 (14.3%)	12 (42.9%)
Refractory CMV disease				0 (0.0%)	
Duration of letermovir use (months)	PP 9.2 (IQR 3.0–11.6) SP 9.5 (IQR 6.4–20.7)	5.9 (range 1.8–25.2)	6 (IQR 2–7)	5 (IQR 3–19)	5.1 ± 4.4 months
Adverse event due to letermovir	PP 5 (19.2%) SP 0 (0.0%)	0 (0.0%)	2 (25%)	0 (0.0%)	
Disseminated VZV/HSV infection			0 (0.0%)	1 (14.3%)	
CMV infection or disease while receiving letermovir	PP 5 (19.2%) SP 10 (62.5%)	8 (47.1%)	3 (37.5%)	0 (0.0%)	5 (17.9%)
Letermovir-resistant CMV/UL56	0 (0.0%)	2 (11.8%)		0 (0.0%)	3 (10.7%)
Continued letermovir use at time of data analysis			5 (62.5%)	3 (42.9%)	

LTx, lung transplant; D, donor; R, recipient; + seropositive; − seronegative; FK-506, tacrolimus; MMF, mycophenolic mofetil; CMV, cytomegalovirus; VZV, varicella-zoster virus; HSV, herpes simplex virus; PP, primary prophylaxis; SP, secondary prophylaxis

Saullo et al. employed letermovir for either primary or secondary prophylaxis in 37 LTx cases, along with an additional four heart-transplant cases [12]. Patients exhibiting CMV mismatch accounted for 56.8% of the total cohort. The predominant rationale for letermovir use was myelosuppression, observed in 96.2% of primary prophylaxis cases and 62.5% of secondary prophylaxis cases. Adverse effects associated with letermovir necessitated prophylaxis cessation in 19.2% of primary prophylaxis instances; however, no interruptions were warranted in cases of secondary prophylaxis. Throughout letermovir administration, 35.7% of cases exhibited minimal CMV replication, which, with the exception of one case of breakthrough CMV infection, did not pose significant concerns. Despite many patients experiencing myelosuppression and being LTx recipients intolerant to valganciclovir, letermovir prophylaxis emerges as an effective agent for suppressing the onset of CMV disease, even in high-risk patients.

Singha et al. documented their experience with letermovir in a cohort of 17 cases. The CMV serology revealed a mismatch in 64.7% of cases, with eight individuals having a prior history of CMV infection [13]. Letermovir was initiated for all subjects because of myelosuppression induced by previous medications, with a distribution of 52.9% for primary prophylaxis and 47.1% for secondary prophylaxis. The authors extensively addressed hematological changes in this report. In the majority of cases, the introduction of letermovir resulted in recovery from myelosuppression. Following the commencement of letermovir, CMV replication was observed in eight individuals, and letermovir-resistant CMV was detected in two cases. No significant adverse effects attributable to letermovir were reported. Even in LTx recipients with severe myelosuppression, letermovir appears to be well tolerated and its use is considered a viable approach for preventing the onset of CMV disease.

Aryal et al. have reported their experiences with letermovir in LTx recipients, initiating its use in 2018, a relatively early period when letermovir had just begun to be used post-HSCT for CMV prophylaxis [14]. Letermovir was administered to eight LTx recipients, with a 75% prevalence of CMV mismatch. Primary prophylaxis was applied in 87.5% of cases, and secondary prophylaxis in 12.5%. Among the cases, one individual progressed to CMV disease and was treated with letermovir. The primary indication for letermovir use was frequently myelosuppression, and one case involved a patient with ganciclovir-resistant CMV. Following letermovir initiation, CMV replication was observed in three individuals, with one experiencing significant CMV disease, while the remaining two did not develop severe illness. The authors concluded that, generally, letermovir is a well-tolerated drug with minor side effects, even in LTx recipients at high risk of CMV disease.

Veit et al. have documented their experience with letermovir, focusing not on its prophylactic use against CMV infection but on its application for the treatment of CMV infection [15]. This research group, acknowledged as pioneers in the field, consistently provides ongoing reports regarding the use of letermovir. Within this report, a cohort of 28 LTx recipients revealed a 53.6% incidence of CMV mismatches. Prior to letermovir introduction, ganciclovir-resistant CMV was present in 42.9% of cases. Following treatment initiation, a rapid resolution of CMV replication was observed in 82.1% of patients, while 17.9% exhibited limited responsiveness to letermovir therapy. Among those with limited responsiveness, half (10.7% of the total) displayed evidence of letermovir resistance. LTx recipients requiring letermovir often exhibit CMV mismatches and are unable to continue ganciclovir because of resistance or side effects, placing them in a high-risk category. In view of the potential for treatment failure or the development of letermovir resistance, close monitoring is imperative for such patients.

## Discussion

We present the experience of using letermovir in LTx recipients in Japan. In all cases within the Japanese cohort, letermovir was administered as a prophylactic measure, with no instances of using letermovir for the treatment of CMV infection or disease. The primary rationale for letermovir administration was myelosuppression, aligning with similar trends observed both domestically and internationally. CMV disease following LTx poses risks of chronic lung allograft dysfunction (CLAD) being developed [16] and serves as an adverse prognostic factor [17, 18]. Prophylactic administration of valganciclovir is an indispensable strategy in post-LTx management to prevent CMV infections [19–22]. However, a proportion of patients cannot continue valganciclovir, presenting a significant challenge, particularly for those at high risk of CMV. To our knowledge, reports on the use of letermovir in LTx are limited to fewer than 100 cases. Nevertheless, it is reasonable to assume that letermovir is more widely used in LTx recipients across transplant centers worldwide. Our findings suggest that, even in high-risk patients facing challenges such as myelosuppression and the inability to continue valganciclovir, letermovir can be safely employed for the prevention of CMV infection or disease without compromising efficacy. While the adoption of letermovir for CMV infection prophylaxis in KTx recipients is anticipated [1], our study highlights the potential utility of letermovir for CMV prophylaxis in LTx recipients unable to continue valganciclovir.

Guidelines recommend the use of valganciclovir for prophylaxis against CMV infection and intravenous ganciclovir for the treatment of CMV disease after solid-organ transplantation [2, 3]. Reports on the use of letermovir in treating CMV infection or disease in LTx recipients are limited, with Veit et al. providing valuable insights into letermovir treatment experiences. Their report indicates a 17.9% incidence of breakthrough CMV infection and a 10.7% occurrence of letermovir-resistant CMV, making the use of letermovir a less unequivocally endorsed therapeutic strategy [15]. However, considering that half of the LTx recipients requiring letermovir treatment exhibited mismatches (D + /R −) and were ganciclovir-resistant, they inherently belong to a high-risk or refractory population, and the treatment efficacy may not be as unfavorable as initially perceived. Conversely, Linder et al. demonstrated positive outcomes using letermovir for CMV infection with a viral load < 1,000 IU/mL; however, in cases with ≥ 1,000 IU/mL, over half of the patients did not achieve CMV clearance [23]. It has been previously reported that the use of letermovir in CMV treatment can induce letermovir resistance, requiring careful consideration in its application [24]. While a head-to-head trial comparing letermovir with ganciclovir in initial CMV treatment would be desirable, a cautious discussion is warranted in view of the outcomes of other organ transplants. Given the limited reports on letermovir for CMV infection treatment in this literature review, drawing definitive conclusions about the agent’s effectiveness following LTx remains challenging.

Numerous transplant centers adopt the prophylactic administration of azole antifungals alongside anti-CMV agents post-LTx. At TUH, all LTx recipients receive lifelong azole antifungal prophylaxis, preferably voriconazole over itraconazole [6]. Letermovir, an inhibitor of cytochrome P450 (CYP) 3A4 and an inducer of CYP2C19, requires special caution when co-administered with calcineurin inhibitors and azole antifungals [25–27]. Tacrolimus is metabolized by CYP3A4, while voriconazole is primarily metabolized by CYP2C19 and strongly inhibits CYP3A4. Among six cases at TUH where Letermovir was used, all patients received tacrolimus, with four also on voriconazole and two on itraconazole. Although blood levels of tacrolimus and voriconazole can be monitored at TUH, letermovir levels remain unmeasurable. It’s worth noting that tacrolimus and voriconazole are simultaneously administered twice daily at TUH under meticulous blood level monitoring. Since voriconazole significantly increases tacrolimus levels by affecting CYP3A4 metabolism, tacrolimus is adjusted to one-fourth the dose upon starting voriconazole. In contrast, letermovir is administered once daily at noon and is not co-administered with the other two drugs. In our experience with all six cases, neither tacrolimus nor voriconazole doses required adjustment pre- or post-letermovir administration, and no significant changes in blood levels were noted. Theoretically, letermovir increases tacrolimus levels through CYP3A4 modulation and decreases voriconazole levels through CYP2C19 modulation, as supported by Phase 1 reports [25, 26]. Interestingly, complicated drug interactions under triple therapy have been reported in hematopoietic stem cell transplant recipients, where letermovir elevates tacrolimus levels while reducing voriconazole levels, ultimately requiring no tacrolimus dose adjustments [28]. Our outcome may partly result from the lack of simultaneous administration of letermovir with the other two drugs. However, given our limited case pool, we cannot conclusively state that avoiding co-administration of letermovir prevented such drug interactions. Ideally, a multicenter study with a substantial case volume would be necessary to verify this. However, such an investigation remains unfeasible due to letermovir’s unapproved status after lung transplantation. It appears imperative to conduct such inquiries at high-volume centers to determine whether off-label letermovir use affects calcineurin inhibitor and azole antifungal blood levels in LTx recipients or to accumulate reports concerning drug interactions under triple therapy from diverse transplant centers worldwide.

In this study, several limitations warrant discussion. Firstly, there is a lack of prospective studies in LTx recipients, akin to those conducted by Limaye AP et al. in KTx recipients [1]. Consequently, the prophylactic efficacy of letermovir against CMV after LTx remains uncertain. Given the higher dosage of immunosuppressive medications in LTx compared to KTx, the elevated risk of CMV infection makes this population likely to benefit from CMV prophylaxis with letermovir. However, this assumption requires validation through clinical trials. A comparative trial, possibly with valganciclovir, would be a reasonable consideration. Additionally, our report indicates that half of the cases were at intermediate risk (D + /R +) for CMV infection. Consequently, the maintenance of CMV-free status may be attributed not only to letermovir administration but also to preexisting immunity. The retrospective nature of the study obscures whether letermovir was necessary in these patients, or if discontinuation of valganciclovir and reduction of mycophenolate mofetil would have sufficed. To address these uncertainties, a prospective trial comparing letermovir use in the intermediate-risk group with an observation group may be insightful.

In conclusion, our study sheds light on the off-label use of letermovir for prophylaxis against CMV infection in LTx recipients, especially those intolerant to valganciclovir. The cases presented illustrate successful outcomes, emphasizing the potential efficacy of letermovir in preventing CMV infection or disease in this high-risk population. In particular, the study advocates a cautious approach, stressing the need for meticulous consideration in incorporating letermovir into the treatment regimen. The safety and tolerability of letermovir were notable, making it a viable alternative when traditional prophylaxes pose challenges.

## Data Availability

The datasets used and/or analyzed during the current study are available from the corresponding author on reasonable request.
