# Correction to: Letermovir prophylaxis for cytomegalovirus in lung‑transplant recipients: a comprehensive study with literature review of off‑label use and real‑world experiences

**DOI:** 10.1007/s10238-024-01358-4

**Published:** 2024-06-01

**Authors:** Takashi Hirama, Yuki Shundo, Toshikazu Watanabe, Akihiro Ohsumi, Tatsuaki Watanabe, Yoshinori Okada

**Affiliations:** 1 Department of Thoracic Surgery, Institute of Development, Aging and Cancer, Tohoku University, Sendai, Miyagi Japan; 2 Division of Organ Transplantation, Tohoku University Hospital, Sendai, Miyagi Japan; 3 Department of Respiratory Medicine, Faculty of Medicine, Fukuoka University, Fukuoka, Fukuoka Japan; 4 Department of Thoracic Surgery, Kyoto University Hospital, Kyoto, Kyoto Japan

Correction to: Clinical and Experimental Medicine (2024) 24:68 10.1007/s10238-024-01330-2

In the original version of this article, the affiliations of the second-last author, “Tatsuaki Watanabe,” were incorrectly listed as affiliation 1, 2, 3, 4, but his correct affiliation should be only 1 “Department of Thoracic Surgery, Institute of Development, Aging and Cancer, Tohoku University, Sendai, Miyagi, Japan”.

And the order of references in the article was incorrect.

The current order of references in the article:

1. Limaye AP, Budde K, Humar A, et al. Letermovir versus valganciclovir for prophylaxis of cytomegalovirus in high-risk kidney transplant recipients: a randomized clinical trial. JAMA. 2023;330(1):33–42. 10.1001/jama.2023.9106.

2. Linder KA, Kovacs C, Mullane KM, et al. Letermovir treatment of cytomegalovirus infection or disease in solid organ and hematopoietic cell transplant recipients. Transpl Infect Dis. 2021;23(4):e13687. 10.1111/tid.13687.

3. Razonable RR, Humar A. Cytomegalovirus in solid organ transplant recipients—guidelines of the american society of transplantation infectious diseases community of practice. Clin Transplant. 2019;33(9):1–23. 10.1111/ctr.13512.

4. Nikkuni E, Hirama T, Hayasaka K, et al. Recovery of physical function in lung transplant recipients with sarcopenia. BMC Pulm Med. 2021;21(1):124. 10.1186/s12890-021-01442-5.

5. Kumata S, Hirama T, Watanabe Y, et al. The fraction of sensitization among lung transplant recipients in a transplant center in Japan. BMC Pulm Med. 2020;20(1):256. 10.1186/s12890-020-01299-0.

6. Hirama T, Tomiyama F, Notsuda H, et al. Outcome and prognostic factors after lung transplantation for bronchiectasis other than cystic fibrosis. BMC Pulm Med. 2021;21(1):261. 10.1186/s12890-021-01634-z.

7. Hirama T, Akiba M, Shundo Y, et al. Efficacy and safety of mRNA SARS-CoV-2 vaccines in lung transplant recipients. J Infect Chemother. 2022;28(8):1153–8. 10.1016/j.jiac.2022.04.019.

8. Ui M, Hirama T, Akiba M, Honda M, Kikuchi T, Okada Y. Cellular and humoral immune responses after a third dose of SARS-CoV-2 mRNA vaccine in lung transplant recipients in Japan. Vaccine. 2023;41(31):4534–40. 10.1016/j.vaccine.2023.06.011.

9. Hirama T, Okada Y. Roles of respirologists in lung transplantation in Japan: narrative review. J Thorac Dis. 2023;15(9):5174–81. 10.21037/jtd-22-1716.

10. Katahira M, Hirama T, Eba S, et al. Impact of postoperative continuous renal replacement therapy in lung transplant recipients. Transplant Direct. 2020;6(6): e562. 10.1097/TXD.0000000000001013.

11. Katada Y, Nakagawa S, Nagao M, et al. Risk factors of breakthrough aspergillosis in lung transplant recipients receiving itraconazole prophylaxis. J Infect Chemother. 2022;28(1):54–60. 10.1016/j.jiac.2021.09.020.

12. Saullo JL, Baker AW, Snyder LD, et al. Cytomegalovirus prevention in thoracic organ transplantation: A single-center evaluation of letermovir prophylaxis. J Heart Lung Transplant. 2022;41(4):508–15. 10.1016/j.healun.2021.12.005.

13. Singha A, Burcham PK, Logan A, et al. Letermovir for cytomegalovirus prophylaxis in lung transplant patients with valganciclovir-induced leukopenia. Transplantology. 2021;2(2):129–39. 10.3390/transplantology2020013.

14. Aryal S, Katugaha SB, Cochrane A, et al. Single-center experience with use of letermovir for CMV prophylaxis or treatment in thoracic organ transplant recipients. Transpl Infect Dis. 2019;21(6): e13166. 10.1111/tid.13166.

15. Veit T, Munker D, Barton J, et al. Letermovir in lung transplant recipients with cytomegalovirus infection: a retrospective observational study. Am J Transplant. 2021;21(10):3449–55. 10.1111/ajt.16718.

16. Paraskeva M, Bailey M, Levvey BJ, et al. Cytomegalovirus replication within the lung allograft is associated with bronchiolitis obliterans syndrome. Am J Transplant. 2011;11(10):2190–6. 10.1111/j.1600-6143.2011.03663.x.

17. Fishman JA, Rubin RH. Infection in organ-transplant recipients. N Engl J Med. 1998;338(24):1741–51. 10.1056/NEJM199806113382407.

18. Rubin RH. The indirect effects of cytomegalovirus infection on the outcome of organ transplantation. JAMA. 1989;261(24):3607–9.

19. Johansson I, Mårtensson G, Nyström U, Nasic S, Andersson R. Lower incidence of CMV infection and acute rejections with valganciclovir prophylaxis in lung transplant recipients. BMC Infect Dis. 2013;13:582. 10.1186/1471-2334-13-582.

20. Hammond SP, Martin ST, Roberts K, et al. Cytomegalovirus disease in lung transplantation: impact of recipient seropositivity and duration of antiviral prophylaxis. Transpl Infect Dis. 2013;15(2):163–70. 10.1111/tid.12036.

21. Finlen Copeland CA, Davis WA, Snyder LD, et al. Long-term efficacy and safety of 12 months of valganciclovir prophylaxis compared with 3 months after lung transplantation: a single-center, long-term follow-up analysis from a randomized, controlled cytomegalovirus prevention trial. J Heart Lung Transplant. 2011;30(9):990–6. 10.1016/j.healun.2011.02.017.

22. Palmer SM, Limaye AP, Banks M, et al. Extended valganciclovir prophylaxis to prevent cytomegalovirus after lung transplantation: a randomized, controlled trial. Ann Intern Med. 2010;152(12):761–9. 10.7326/0003-4819-152-12-201006150-00003.

23. Cherrier L, Nasar A, Goodlet KJ, Nailor MD, Tokman S, Chou S. Emergence of letermovir resistance in a lung transplant recipient with ganciclovir-resistant cytomegalovirus infection. Am J Transplant. 2018;18(12):3060–4. 10.1111/ajt.15135.

24. McCrea JB, Macha S, Adedoyin A, et al. Pharmacokinetic drug-drug interactions between letermovir and the immunosuppressants cyclosporine, tacrolimus, sirolimus, and mycophenolate mofetil. J Clin Pharmacol. 2019;59(10):1331–9. 10.1002/jcph.1423.

25. Marshall WL, McCrea JB, Macha S, et al. Pharmacokinetics and tolerability of letermovir coadministered with azole antifungals (posaconazole or voriconazole) in healthy subjects. J Clin Pharmacol. 2018;58(7):897–904. 10.1002/jcph.1094.

26. Nakashima T, Inamoto Y, Fukushi Y, et al. Drug interaction between letermovir and voriconazole after allogeneic hematopoietic cell transplantation. Int J Hematol. 2021;113(6):872–6. 10.1007/s12185-021-03105-x.

27. Hikasa S, Shimabukuro S, Osugi Y, et al. Tacrolimus Concentration after Letermovir Initiation in Hematopoietic Stem Cell Transplantation Recipients Receiving Voriconazole: A Retrospective. Observational Study Int J Med Sci. 2020;17(7):859–64. 10.7150/ijms.42011.

28. Kotton CN, Kumar D, Caliendo AM, et al. The third international consensus guidelines on the management of cytomegalovirus in solid organ transplantation. Transplant Publ online. 2018. 10.1097/TP.0000000000002191.

The order of references should have been.

1. Limaye AP, Budde K, Humar A, et al. Letermovir vs Valganciclovir for Prophylaxis of Cytomegalovirus in High-Risk Kidney Transplant Recipients: A Randomized Clinical Trial. JAMA. 2023;330(1):33–42. 10.1001/jama.2023.9106.

2. Razonable RR, Humar A. Cytomegalovirus in solid organ transplant recipients—Guidelines of the American Society of Transplantation Infectious Diseases Community of Practice. Clin Transplant. 2019;33(9):1–23. 10.1111/ctr.13512.

3. Kotton CN, Kumar D, Caliendo AM, et al. The Third International Consensus Guidelines on the Management of Cytomegalovirus in Solid Organ Transplantation. Transplantation. Published online 2018. 10.1097/TP.0000000000002191.

4. Nikkuni E, Hirama T, Hayasaka K, et al. Recovery of physical function in lung transplant recipients with sarcopenia. BMC Pulm Med. 2021;21(1):124. 10.1186/s12890-021-01442-5.

5. Kumata S, Hirama T, Watanabe Y, et al. The fraction of sensitization among lung transplant recipients in a transplant center in Japan. BMC Pulm Med. 2020;20(1):256. 10.1186/s12890-020-01299-0.

6. Hirama T, Tomiyama F, Notsuda H, et al. Outcome and prognostic factors after lung transplantation for bronchiectasis other than cystic fibrosis. BMC Pulm Med. 2021;21(1):261. 10.1186/s12890-021-01634-z.

7. Hirama T, Akiba M, Shundo Y, et al. Efficacy and safety of mRNA SARS-CoV-2 vaccines in lung transplant recipients. J Infect Chemother. 2022;28(8):1153–1158. 10.1016/j.jiac.2022.04.019.

8. Ui M, Hirama T, Akiba M, Honda M, Kikuchi T, Okada Y. Cellular and humoral immune responses after a third dose of SARS-CoV-2 mRNA vaccine in lung transplant recipients in Japan. Vaccine. 2023;41(31):4534–4540. 10.1016/j.vaccine.2023.06.011.

9. Hirama T, Okada Y. Roles of respirologists in lung transplantation in Japan: narrative review. J Thorac Dis. 2023;15(9):5174–5181. 10.21037/jtd-22-1716.

10. Katahira M, Hirama T, Eba S, et al. Impact of Postoperative Continuous Renal Replacement Therapy in Lung Transplant Recipients. Transplant Direct. 2020;6(6):e562. 10.1097/TXD.0000000000001013.

11. Katada Y, Nakagawa S, Nagao M, et al. Risk factors of breakthrough aspergillosis in lung transplant recipients receiving itraconazole prophylaxis. J Infect Chemother. 2022;28(1):54–60. 10.1016/j.jiac.2021.09.020.

12. Saullo JL, Baker AW, Snyder LD, et al. Cytomegalovirus prevention in thoracic organ transplantation: A single-center evaluation of letermovir prophylaxis. J Heart Lung Transplant. 2022;41(4):508–515. 10.1016/j.healun.2021.12.005.

13. Singha A, Burcham PK, Logan A, et al. Letermovir for Cytomegalovirus Prophylaxis in Lung Transplant Patients with Valganciclovir-Induced Leukopenia. Transplantology. 2021;2(2):129–139. 10.3390/transplantology2020013.

14. Aryal S, Katugaha SB, Cochrane A, et al. Single-center experience with use of letermovir for CMV prophylaxis or treatment in thoracic organ transplant recipients. Transpl Infect Dis. 2019;21(6):e13166. 10.1111/tid.13166.

15. Veit T, Munker D, Barton J, et al. Letermovir in lung transplant recipients with cytomegalovirus infection: A retrospective observational study. Am J Transplant. 2021;21(10):3449–3455. 10.1111/ajt.16718.

16. Paraskeva M, Bailey M, Levvey BJ, et al. Cytomegalovirus replication within the lung allograft is associated with bronchiolitis obliterans syndrome. Am J Transplant. 2011;11(10):2190–2196. 10.1111/j.1600-6143.2011.03663.x

17. Fishman JA, Rubin RH. Infection in organ-transplant recipients. N Engl J Med. 1998;338(24):1741–1751. 10.1056/NEJM199806113382407.

18. Rubin RH. The indirect effects of cytomegalovirus infection on the outcome of organ transplantation. JAMA. 261(24):3607–3609.

19. Johansson I, Mårtensson G, Nyström U, Nasic S, Andersson R. Lower incidence of CMV infection and acute rejections with valganciclovir prophylaxis in lung transplant recipients. BMC Infect Dis. 2013;13:582. 10.1186/1471-2334-13-582.

20. Hammond SP, Martin ST, Roberts K, et al. Cytomegalovirus disease in lung transplantation: impact of recipient seropositivity and duration of antiviral prophylaxis. Transpl Infect Dis. 2013;15(2):163–170. 10.1111/tid.12036.

21. Finlen Copeland CA, Davis WA, Snyder LD, et al. Long-term efficacy and safety of 12 months of valganciclovir prophylaxis compared with 3 months after lung transplantation: a single-center, long-term follow-up analysis from a randomized, controlled cytomegalovirus prevention trial. J Heart Lung Transplant. 2011;30(9):990–996. 10.1016/j.healun.2011.02.017.

22. Palmer SM, Limaye AP, Banks M, et al. Extended valganciclovir prophylaxis to prevent cytomegalovirus after lung transplantation: a randomized, controlled trial. Ann Intern Med. 2010;152(12):761–769. 10.7326/0003-4819-152-12-201006150-00003.

23. Linder KA, Kovacs C, Mullane KM, et al. Letermovir treatment of cytomegalovirus infection or disease in solid organ and hematopoietic cell transplant recipients. Transpl Infect Dis. 2021;23(4):e13687. 10.1111/tid.13687.

24. Cherrier L, Nasar A, Goodlet KJ, Nailor MD, Tokman S, Chou S. Emergence of letermovir resistance in a lung transplant recipient with ganciclovir-resistant cytomegalovirus infection. Am J Transplant. 2018;18(12):3060–3064. 10.1111/ajt.15135.

25. McCrea JB, Macha S, Adedoyin A, et al. Pharmacokinetic Drug-Drug Interactions Between Letermovir and the Immunosuppressants Cyclosporine, Tacrolimus, Sirolimus, and Mycophenolate Mofetil. J Clin Pharmacol. 2019;59(10):1331–1339. 10.1002/jcph.1423.

26. Marshall WL, McCrea JB, Macha S, et al. Pharmacokinetics and Tolerability of Letermovir Coadministered With Azole Antifungals (Posaconazole or Voriconazole) in Healthy Subjects. J Clin Pharmacol. 2018;58(7):897–904. 10.1002/jcph.1094.

27. Nakashima T, Inamoto Y, Fukushi Y, et al. Drug interaction between letermovir and voriconazole after allogeneic hematopoietic cell transplantation. Int J Hematol. 2021;113(6):872–876. 10.1007/s12185-021-03105-x.

28. Hikasa S, Shimabukuro S, Osugi Y, et al. Tacrolimus Concentration after Letermovir Initiation in Hematopoietic Stem Cell Transplantation Recipients Receiving Voriconazole: A Retrospective, Observational Study. Int J Med Sci. 2020;17(7):859–864. 10.7150/ijms.42011.

The original article has been corrected.

